# Supplementary material for: Retention time prediction for chromatographic enantioseparation by quantile geometry-enhanced graph neural network
Source: Nat Commun. 2023 May 29;14:3095. doi: 10.1038/s41467-023-38853-3 (PMC10227049; doi:10.1038/s41467-023-38853-3)
Supplement: Supplementary file 1 — Supplementary Information [file 41467_2023_38853_MOESM1_ESM.pdf]

Supplementary Information for Retention Time Prediction for Chromatographic Enantioseparation by Quantile Geometry-based Graph Neural Network

Hao Xu[a,b], Jinglong Lin[a], Dongxiao Zhang[c,d,*], Fanyang Mo[a,e,*]

[a *] *School of Materials Science and Engineering, Peking University, Beijing, 100871, P. R. China.*

[b] *BIC-ESAT, ERE, and SKLTCS, College of Engineering, Peking University, Beijing, 100871, P. R. China.*

[c] *Eastern Institute for Advanced Study, Eastern Institute of Technology, Ningbo, Zhejiang 315200, P. R. China.*

[d] *Department of Mathematics and Theories, Peng Cheng Laboratory, Shenzhen 518000, Guangdong, P. R. China.*

[e] *AI for Science (AI4S)-Preferred Program, Peking University Shenzhen Graduate School, Shenzhen, 518055, China.*

Table of Contents

Supplementary Notes **2**

1. Supplementary information for the CMRT dataset 2
    - 1.1 Statistical analysis of the CMRT dataset 2
    - 1.2 Visualization of the dataset 3
2. Supplementary experiments and demonstration 3
    - 2.1 Result of cross-validation 3
    - 2.2 The ablation experiment for QGeoGNN 3
    - 2.3 The influence of column features in the multiple columns prediction 3
    - 2.4 Decision of S_p threshold 4
    - 2.5 Prediction for unfamiliar molecules 5

Supplementary Figures and Tables **6**

Supplementary Notes

1. Supplementary information for the CMRT dataset

1.1 Statistical analysis of the CMRT dataset

In this section, additional analysis of the CMRT dataset is provided to reveal some interesting aspects of the field of asymmetric catalysis. Firstly, the distribution of the corresponding authors of these articles is displayed in the way of a word cloud (Supplementary Fig. 2a) and a pie plot (Supplementary Fig. 2b). From the figure, it can be seen that the literature is mainly chosen from those of Chinese scientists that conduct asymmetric catalysis well. Among them, Zhang Wanbin from Shanghai Jiao Tong University (SJTU) contributes the most chiral molecules (18.9%) in our dataset, who has long been committed to the design and synthesis of chiral catalysts. Next, the chiral molecule data contributed by each article are counted. On average, every article contributes 40 data on chiral molecules. However, some articles have discovered a large number of chiral molecules, which is demonstrated in Supplementary Fig. 2c. It can be found that up to 256 chiral molecules were contributed in one article. The median data volume contributed in each article is 36. These data can reflect the general situation of articles published in chiral separation to some extent.

From the distribution of column types that is illustrated in Fig. 3a in the manuscript. It is found that ADH, ODH, IA, and IC are the four columns with the largest data volume. Among them, the frequency of utilization of ADH and ODH columns is far more than other columns, which reflects the broader usage of these two column types. Meanwhile, it is also discovered that high-efficiency columns like ADH, ODH, ASH, and OJH are more popular with fine particle size and high column efficiency. Moreover, chemically bonded series columns that appeared in recent years like IC and IA series columns have attracted more and more attention since it is not limited by elution solvent and is more efficient.

From the aspect of chiral recognition, three factors, including the substrate (cellulose or amylose), the substituents, and the packing material size, mainly account for the difference between diversified HPLC columns to a great extent. For example, AD, AD3, and ADH have the same substrates and substituents while the packing material size is different. To better investigate the distribution of these factors in the dataset, we classify data under different columns according to the three factors and conduct statistical analysis and the results are depicted in Supplementary Fig. 2d, e, and f. From Supplementary Fig. 2d, it can be found that the data volume from HPLC columns with amylose-based chiral stationary phases (CSPs) is nearly the same as that from HPLC columns with cellulose-based CSPs. It is a very interesting phenomenon since amylose and cellulose are enantiomers themselves, which means that they are sensitive to different chiral molecules. The results provide a perfect reflection of the symmetry of chirality because the separation of a large number of chiral molecules on this pair of enantiomers (amylose and cellulose) shows a perfect balance. From Supplementary Fig. 2e, the distribution of packing material sizes utilized in the columns is illustrated, it can be seen that the HPLC column with a moderate packing material size is mostly utilized (80.3% for 5μm). It may account for the fact a moderate packing material size brings a suitable retention time for chiral separation. In the columns utilized in this work, there are 7 types of substituents (Supplementary Fig. 2g). Here, the frequency of these substituents is counted and provided in Supplementary Fig. 2f. It can be seen that substituent A is the most frequently used substituent among all CSPs. We hope that these statistical outcomes may assist the researchers in

better understanding the usage situation of various columns and their influencing factors, and provide some basic references for their selection of HPLC columns.

1.2 Visualization of the dataset

To help readers better understand the molecules contained in the CMRT dataset constructed in this work, we provide the visualization of some enantiomers in the dataset as examples in Supplementary Fig. 3. Considering that there are 11,720 pairs of enantiomers, the pages cannot contain the visualization of all enantiomers, we only provide 10 pairs with 20 chiral molecules for example here. In the manuscript, we have published the dataset in the open source and anyone can visualize the structure of the molecules in the dataset via provided SMILES.

2. Supplementary experiments and demonstration

2.1 Result of cross-validation

In this work, the dataset is split randomly into the training, validating, and testing datasets. The randomness may have an impact on the outcome. Therefore, in this section, cross-validation is conducted to avoid the influence of randomness. In the manuscript, the dataset is split into the training dataset, validating dataset, and testing dataset by 90/5/5. For the cross-validation, the model is trained using a different 5% testing dataset successively until the entire dataset is tested. Therefore, 20 models with different testing datasets are trained independently. For ADH, ODH, IA, and IC columns that are taken as examples in the manuscript, all testing data for each column type in these models are depicted in Supplementary Fig. 5. The mean average error (MAE), median relative error (MRE), and R^2 are also calculated to examine the synthetical performance of the QGeoGNN. From the figure, it is discovered that although the performance of the model varies with different testing datasets, the synthetical predictive accuracy keeps relatively high, which confirms the ability of the proposed QGeoGNN to learn the molecular structure–retention relationship well in different column types.

2.2 The ablation experiment for QGeoGNN

The ablation experiment is usually conducted to observe the contribution of each element to the algorithm. There are several important elements in the QGeoGNN, including the added experimental condition (i.e., the elution proportion) and the incorporated molecular descriptors. In this section, the ablation experiment is conducted on QGeoGNN to discover the function of the above-mentioned elements. The single-column prediction with the ADH column is investigated here as an example. We use GNN as the baseline and add different combinations of elements to observe the change in model performance. The results are provided in Supplementary Table 1. It can be seen that the proportion is of great significance to the prediction accuracy, while the descriptors only improve the performance of the model to some extent.

2.3 The influence of column features in the multiple columns prediction

In the multi-column prediction, the domain knowledge about the HPLC experiment is combined with the proposed QGeoGNN by incorporating the column features including packing material size, substrates, and substituents. The addition of these column features contributes to a synthetic

prediction model that can handle a variety of columns simultaneously. In this section, the influence of column features in the multi-column prediction is examined. For comparison, another model is trained without incorporating these column features while other conditions are kept the same. The comparison between models with and without column features is displayed in Supplementary Fig. 6. From the figure, it can be seen that the predictive accuracy decreases a lot without incorporating column features since the R^2 decreases from 0.702 to 0.635 and the RMSE increases from 3.40 to 3.75, which implies that the column features play an important role in multi-column prediction. Meanwhile, it is found that the prediction model remains a certain prediction ability without column features, which means that the retention time is not only influenced by the column but also influenced by the molecular property.

2.4 Decision of S_p threshold

Although the chromatographic separation probability S_p can measure the probability of enantioseparation, a threshold is required to give an ultimate judgment that the enantiomers are separable or inseparable. In order to make the threshold more efficient and precise, we use 84 experimental data generated in the laboratory as the real data to search for the most proper threshold. In convention, the receiver operating characteristic (ROC) curve is employed to find the best threshold for a classifier, which balances the trade-off between the accuracy of the positive and negative classes by True Positive Rate (TPR) and False Positive Rate (FPR). The point that is closest to the top-left corner of the ROC curve is usually regarded as the most proper threshold. However, it is worth noting that the ROC curve requires a sufficient number of samples to generate a smooth curve. If the sample size is relatively small, the ROC curve may be presented in polylines instead of smoothed curves, as shown in Supplementary Fig. 8. In such cases, the low resolution for thresholds may pose limitations to the interpretation of the curve.

Therefore, we have explored alternative methods for selecting the best threshold, where the overall accuracy of our enantioseparation prediction model is evaluated, which takes into account both the accuracy of predicting separable and inseparable enantiomers. The definition is written as:

$$\begin{aligned} P &= P_{separable} + P_{inseparable} \\ &= \frac{TP}{N_{separable}} + \frac{TN}{N_{inseparable}}, \end{aligned} \qquad \text{(S.1)}$$

where $P_{separable}$ and $P_{inseparable}$ are the accuracies of predicting separable and inseparable enantiomers; TP (True Positive) refers to the number of separable enantiomers to be correctly predicted as separable while TN (True Negative) refers to the number of inseparable enantiomers to be correctly predicted as inseparable. $N_{separable}$ and $N_{inseparable}$ are the numbers of separable enantiomers and inseparable enantiomoers, respectively. Therefore, the overall accuracy P evaluates both accuracies of the model for predicting separable and inseparable enantiomers. A higher P means a more balanced performance of prediction. The calculated P in different thresholds is provided in Supplementary Fig. 9. It can be seen that P reaches the maximum when the threshold is 0.38. Of note, the P remains relatively high across the entire range of S_p threshold values considered since it takes both accuracies of separable and inseparable enantiomers into account. Specifically, when the threshold is set relatively high, the $P_{separable}$ will decrease and $P_{inseparable}$ will increase, which makes

the overall accuracy maintains high. Similar to the ROC curve, the overall accuracy defined in this section balances the accuracy of the positive and negative classes. Of note, the best threshold found by the ROC is 0.39 (Supplementary Fig. 8), which is close to the 0.38 found in this section, which also proves the effectiveness of the defined overall accuracy. Overall, in this work, the threshold is decided to be 0.38, which means that enantiomers with S_p larger than 0.38 will be seen as separable.

2.5 Prediction for unfamiliar molecules

In the manuscript, the QGeoGNN is proven to have a satisfactory predictive ability on the established CMRT dataset. Considering that there exist innumerable varieties of compounds in this world, the extensibility of QGeoGNN to completely unfamiliar molecules is of great importance. Therefore, we conducted HPLC analysis in the laboratory and obtained 136 experimental data of newly generated enantiomers that is different from the molecules in the dataset under different experimental conditions. The prediction model trained on the CMRT dataset is employed to predict the retention time of these molecules to investigate the extensibility of QGeoGNN. The result is shown in Supplementary Fig. 10a. It is discovered that although the predictive performance decreases to some extent, the prediction of *RT* value is generally accurate. Furthermore, we also calculate the S_p of each enantiomer to identify the ability of QGeoGNN to facilitate chromatographic enantioseparation when faced with new molecules. The violin plot of the calculated S_p is illustrated in Supplementary Fig. 10b. Compared with the violin plot of calculated S_p inside the CMRT dataset (Fig. 6b in the manuscript), the violin plot of unfamiliar molecules is more uniformly distributed, which means that these enantiomers are more difficult to determine whether they can be separated under given conditions. To calculate the accuracy of separation prediction, we regard those enantiomers with S_p>0.38 as separable, and the accuracy only reaches 73.0%, which has a large space for improvement. Of course, this result is understandable, because neural networks have limited prediction ability for extrapolated data, so predicting completely unfamiliar molecules is a challenging task essentially. We believe that QGeoGNN will have a good prediction ability for various molecules as more data are collected.

Supplementary Figures and Tables

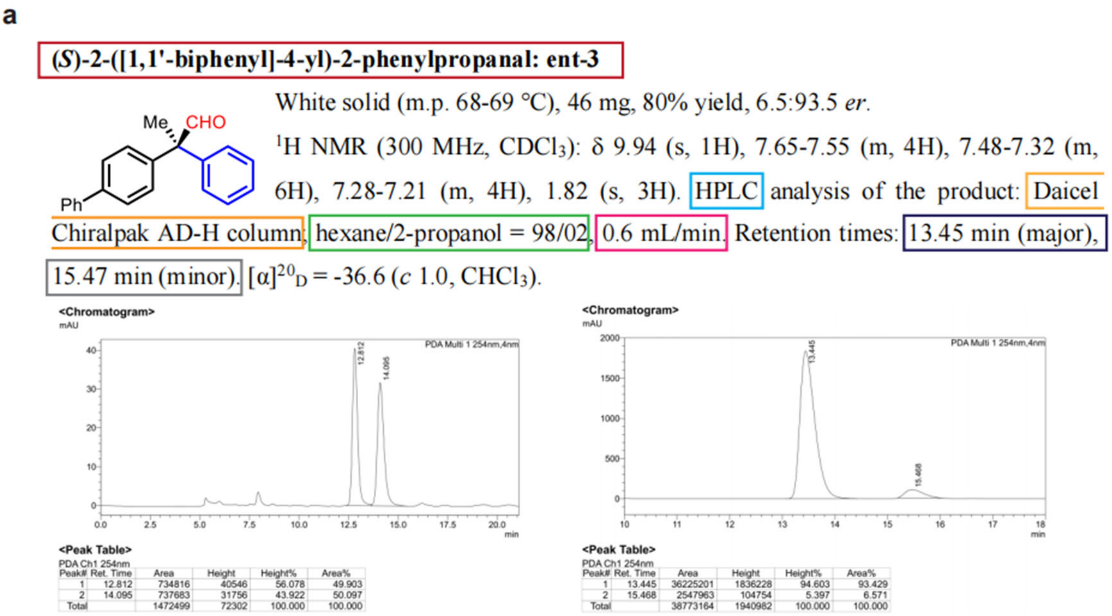

Peak#	Ret. Time	Area	Height	Height%	Area%
1	12.812	734816	40546	56.078	49.903
2	14.095	737683	31756	43.922	50.097
Total		1472499	72302	100.000	100.000

Peak#	Ret. Time	Area	Height	Height%	Area%
1	13.445	36225201	1836228	94.603	93.429
2	15.468	2547963	104754	5.397	6.571
Total		38773164	1940982	100.000	100.000

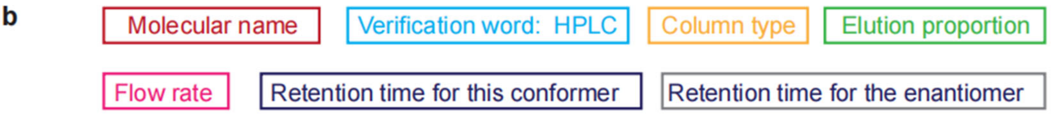

Supplementary Figure 1. An example of the experimental result reported in the literature and extracted information. a, The style of a typical experimental report when separating chiral molecules in the asymmetric catalysis literature. **b**, The extracted keywords that are employed to construct the dataset. AU refers to the absorbance unit.

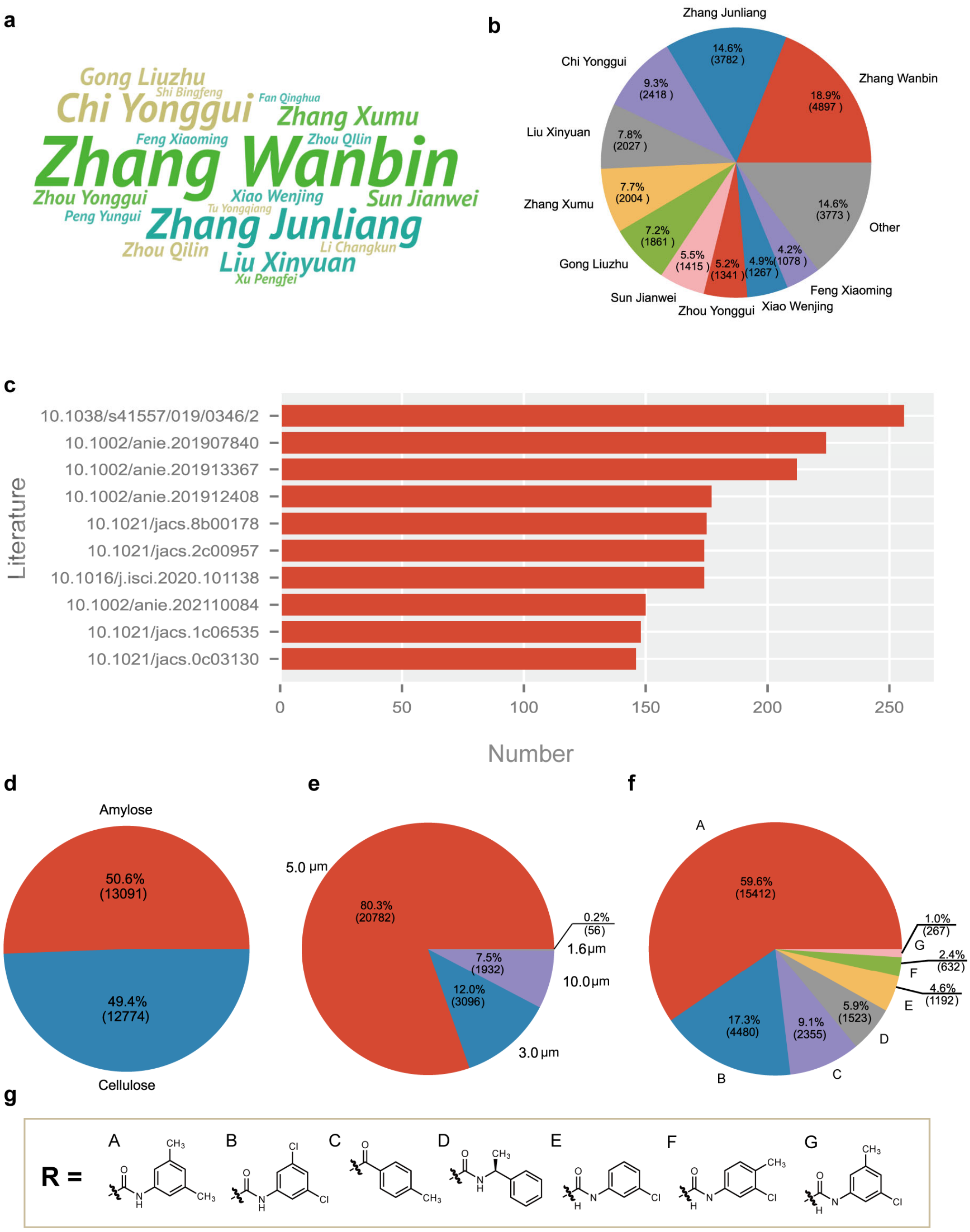

Supplementary Figure 2. The statistical analysis of the CMRT dataset. a, A word cloud of the corresponding authors for literature in the CMRT dataset. **b,** The pie plot of the corresponding authors for literature in the CMRT dataset. **c,** Top 10 articles reporting the largest number of enantiomers. The y-axis is the doi of the article. **d,** The pie plot of the distribution of substrates utilized in the high-performance liquid chromatography (HPLC) columns. **e,** The pie plot of the packing material size utilized in the HPLC columns. **f,** The pie plot of the substituents utilized in the HPLC columns. **g**, The types of substituents.

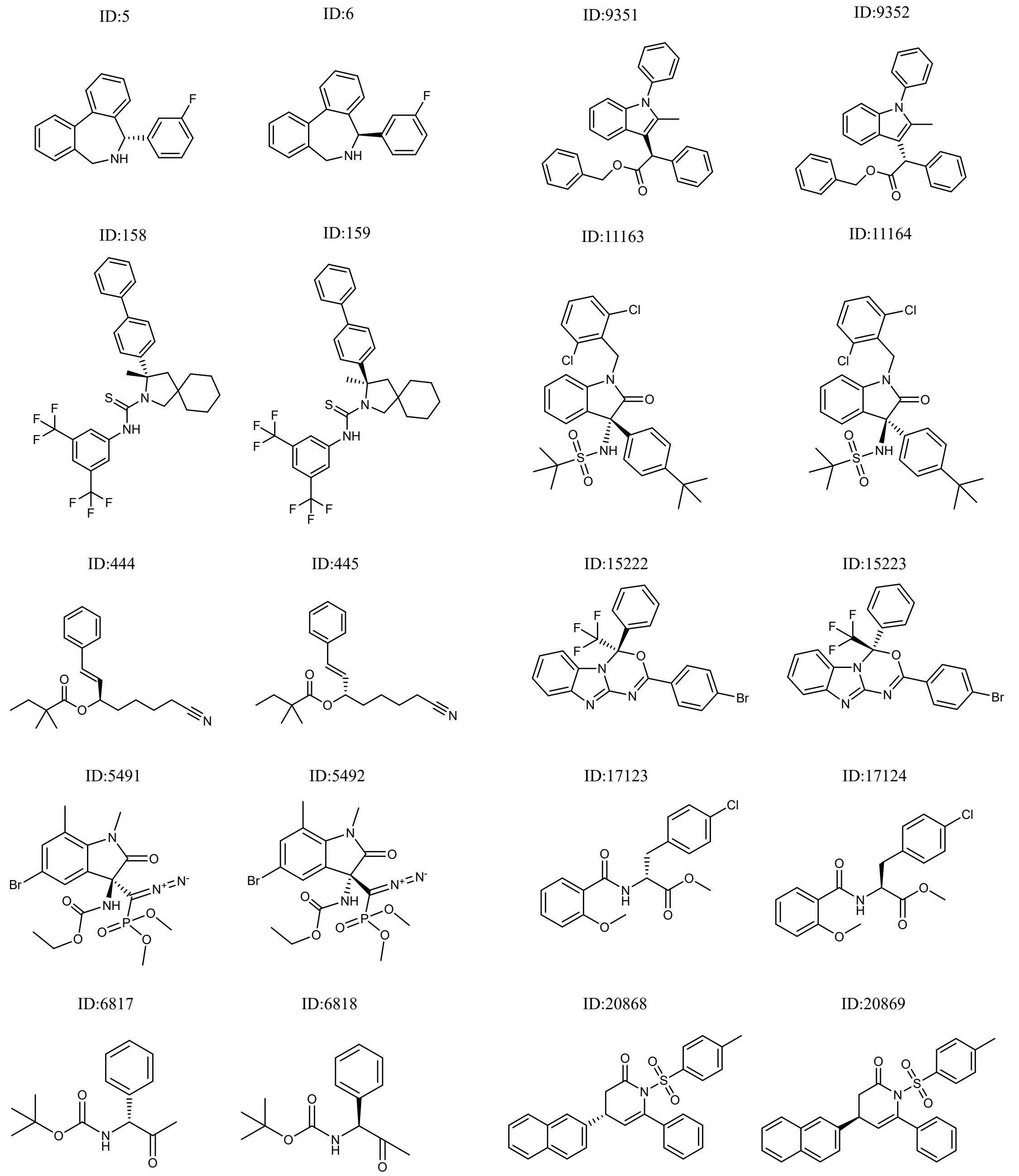

Supplementary Figure 3. The visualization of some enantiomers in the CMRT dataset. 10 pairs with 20 chiral molecules are illustrated here. The ID is the index of the molecule in the CMRT dataset.

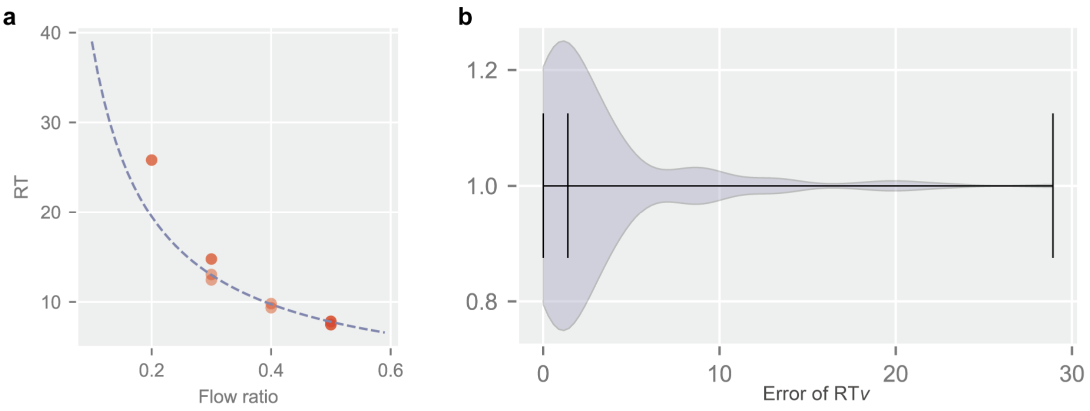

Supplementary Figure 4. Experimental verification of chromatographic process equation (Eq. 1). (a) The red dots are the experimental observation, and the pink dotted line is the fitted curve from the chromatographic process equation. **(b)** The violin plot of the error distribution of the calculated RTv (396 data), and the black vertical lines mean the minimum, median, and maximum values, respectively. RT refers to the retention time while RTv refers to the RT×v where v is the flow rate.

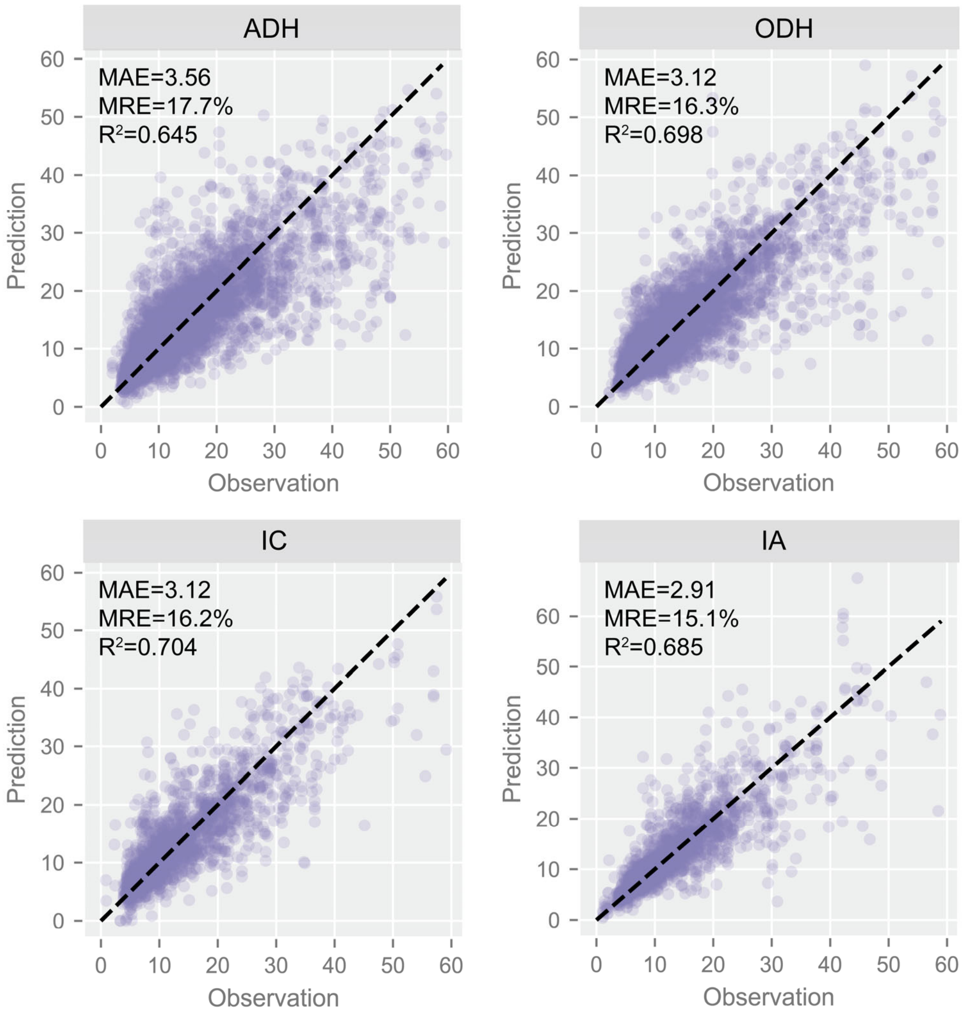

Supplementary Figure 5. The results of cross-validation. Observation *versus* prediction for the proposed QGeoGNN in cross-validation. All testing data in 20 independent models are shown in plots. The dashed line is the $y = x$ line. The measurements are the mean average error (MAE), median relative error (MRE), and coefficient of determination (R^2).

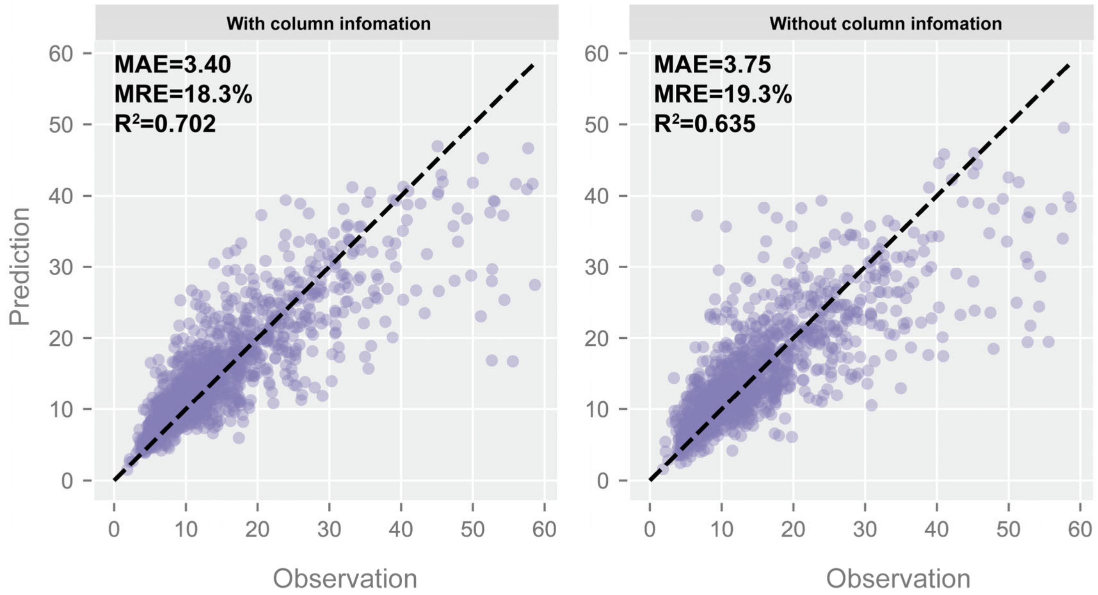

Supplementary Figure 6. The comparison between the model trained with and without column information. Observation versus prediction for the proposed QGeoGNN to predict out-of-sample molecules. Only testing data are shown in plots. The dashed line is the $y = x$ line. The measurements are the mean average error (MAE), median relative error (MRE), and coefficient of determination (R^2).

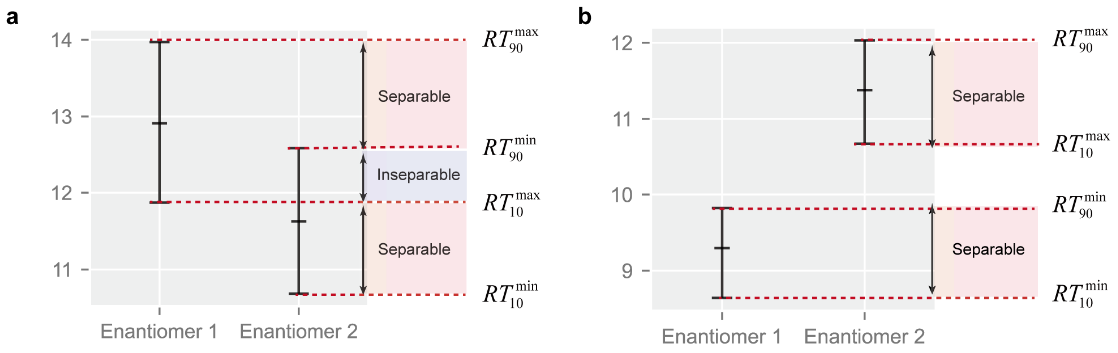

Supplementary Figure 7. Examples for calculating separation probability S_p in two typical scenarios. The red region is predicted to be separable and the blue region is predicted to be inseparable. The lines in the error bar refer to 90th quantile, predicted RT, and 10th quantile, respectively, which are predicted by the model. Here, RT_{90}^{max} and RT_{90}^{min} are the maximum and minimum of 90th percentiles for both enantiomers, RT_{10}^{max} and RT_{10}^{min} are the maximum and minimum of 10th percentiles, respectively.

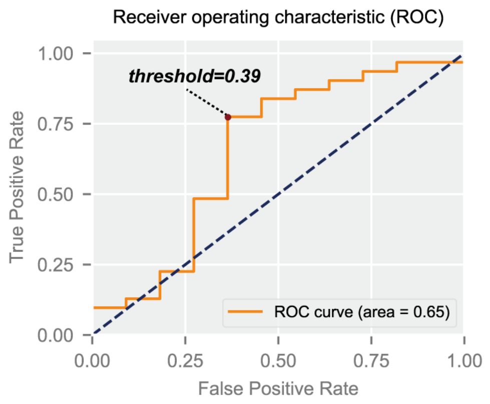

Supplementary Figure 8. The ROC curve for the model to predict the enantioseparation of real data. The threshold refers to the S_p threshold which is decided by the point that is closest to the top-left corner of the ROC curve.

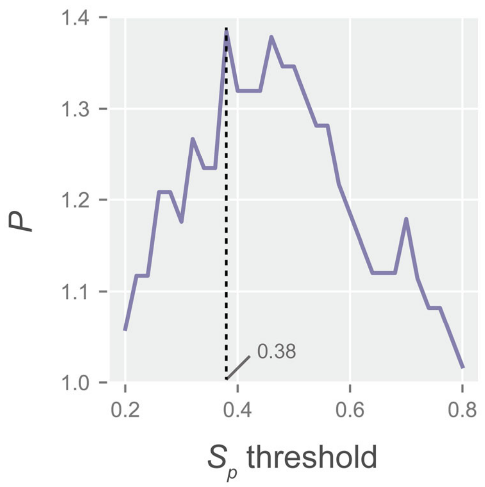

Supplementary Figure 9. Overall accuracy *P* with different S_p thresholds. Here, *P* is the overall accuracy of our enantioseparation prediction model, and S_p is the separation probability. The best threshold is 0.38 which is shown in a dashed line.

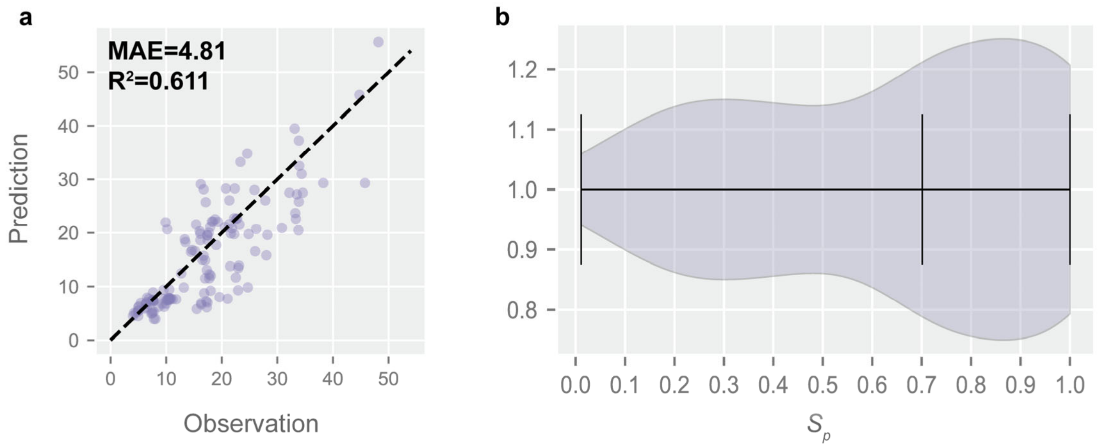

Supplementary Figure 10. Prediction for unfamiliar molecules. a, Observation versus prediction for the proposed QGeoGNN to predict completely unfamiliar enantiomers. Only testing data are shown in plots. The dashed line is the $y = x$ line. **b,** The violin plot of the distribution of calculated S_p for unfamiliar enantiomers (136 data). The measurements are the mean average error (MAE) and coefficient of determination (R^2). S_p is the separation probability.

Supplementary Table 1. The mean average error (MSE), root mean average error (RMSE), and coefficient of determination (R^2) of the single-column prediction in the ablation experiment. The baseline is the model without adding the descriptors and elution proportion. +Descriptors refers to the model only adding relevant descriptors. +Proportion refers to the model only adding the elution proportion.

	**Baseline**	**+Descriptors**	**+Proportion**	**+Descriptors & Proportion**
**MSE**	39.88	36.22	24.12	17.72
**RMSE**	6.315	6.018	4.910	4.210
R^2	0.5353	0.5673	0.7015	0.7240
